# Korean Red Ginseng Potentially Improves Maintaining Antibodies after COVID-19 Vaccination: A 24-Week Longitudinal Study

**DOI:** 10.3390/nu15071584

**Published:** 2023-03-24

**Authors:** Jihyun Yoon, Byoungjin Park, Heejung Kim, Seungjun Choi, Donghyuk Jung

**Affiliations:** 1 Department of Family Medicine, Yonsei University College of Medicine, Seoul 03722, Republic of Koreabjpark96@yuhs.ac (B.P.); 2 Department of Laboratory Medicine, Yonsei University College of Medicine, Seoul 03722, Republic of Korea; 3 Department of Laboratory Medicine, Yongin Severance Hospital, Yonsei University College of Medicine, Yongin 16995, Republic of Korea

**Keywords:** Korean Red Ginseng, COVID-19, vaccination, humoral immunity, immunomodulator

## Abstract

Despite the effectiveness and safety of COVID-19 vaccines, vaccine-induced responses decline over time; thus, booster vaccines have been approved globally. In addition, interest in natural compounds capable of improving host immunity has increased. This study aimed to examine the effect of Korean Red Ginseng (KRG) on virus-specific antibodies after COVID-19 vaccination. We conducted a 24 week clinical pilot study of 350 healthy subjects who received two doses of the COVID-19 vaccine and a booster vaccination (third dose). These subjects were randomized 1:2 to the KRG and control groups. We evaluated antibody response five times: just before the second dose (baseline), 2 weeks, 4 weeks, 12 weeks after the second dose, and 4 weeks after the third dose. The primary endpoints were changes in COVID-19 spike antibody titers and neutralizing antibody titers. The antibody formation rate of the KRG group was sustained higher than that of the control group for 12 weeks after the second dose. This trend was prominently observed in those above 50 years old. We found that KRG can help to increase and maintain vaccine response, highlighting that KRG could potentially be used as an immunomodulator with COVID-19 vaccines.

## 1. Introduction

Coronavirus disease 2019 (COVID-19), which is caused by infection with severe acute respiratory syndrome coronavirus 2 (SARS-CoV-2), has caused significant morbidity and mortality worldwide and an ongoing global public health crisis [1]. There are limited safe and specific pharmaceutical agents or recommendations to prevent or treat COVID-19. Therefore, vaccines against SARS-CoV-2 infection that stimulate the host’s immune system have been considered the best way to control the pandemic [2]. However, because of emerging hurdles such as new variants of SARS-CoV-2 that overcome vaccine-induced host immunity [3] and waning of vaccine-induced neutralizing antibodies (anti-N-Ab) over time [4]. Booster vaccination has been approved for fully vaccinated individuals, because it have been shown to enhance protection against SARS-CoV-2 compared with only a primary vaccination series [5,6].

Recent studies have suggested that the effectiveness of COVID-19 boosters also tends to dwindle over time, with waning occurring 5–9 weeks after a booster vaccination [3,7]. Vaccination is a partial solution to preventing early infection and severe illness, and continued optimization of plans for primary prevention of COVID-19 in vaccinated persons is needed [8]. There has been a rising interest in immunomodulators that enhance host immunity to the virus and support the resilience of an infected host until a cure can be developed [9].

Korean Red Ginseng (KRG), derived from *Panax ginseng* Meyer and extracted by steaming, is an herbal medicine with well-defined beneficial effects for the prevention and treatment of various diseases [10,11]. Human studies and animal experiments have shown that KRG plays an essential role as an immunomodulatory agent by improving the host immune response [12,13,14,15]. In addition, KRG has been suggested to increase recovery rates and reduce mortality from viral infections [16]. KRG also increases protective effects against infections, including influenza [17,18], and clinical reports suggest favorable effects of KRG in acute respiratory illness [19]. Moreover, some evidence has suggested that KRG can improve vaccine efficacy. For example, studies have reported that KRG helped reduce inflammation and improve the efficacy of pneumococcal vaccines by inhibiting reactive oxygen species production [20]. KRG improves antibody formation after influenza vaccination, clinically protecting against influenza virus infection [21].

However, no study has examined whether KRG affects COVID-19 vaccine-induced immune response and protection. Therefore, this study aimed to investigate the effect of KRG on the humoral response of hosts who received two doses of COVID-19 vaccines and a booster vaccination (third dose) in Korea.

## 2. Materials and Methods

### 2.1. Study Design and Participants

**Figure 1:**
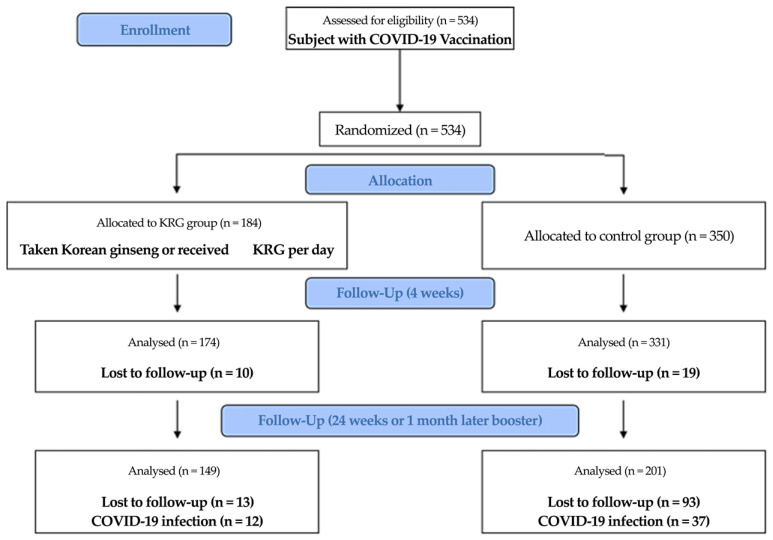
Flow chart for participant selection.

This longitudinal study evaluated KRG intake’s effect on COVID-19 antibodies after vaccination in Korean adults (Clinical Research Information Service, KCT0007342). The study was approved by the Institutional Review Board of Yongin Severance Hospital (IRB no. 9-2021-0101) and was conducted in compliance with the Declaration of Helsinki. Written informed consent was provided by all patients prior to participation. Sample size was calculated based on the following assumptions: the effect size (cohen’s d) 0.25, type I error of 0.05, power of 80%, and 1:2 ratio (KRG/control ratio). The number of subjects to be enrolled was determined to be 190 for the KRG group and 380 for the control group. The originally intended total sample size was 570, but the required number of enrollments was not achieved within the given study period. The KRG dose used in this study was 3 g of KRG tablet/day, containing ginsenoside, Rb1 (8.03 mg/g), Rb2 (2.80 mg/g), Rg3 (2.50 mg/g), Rg1 (1.18 mg/g), Rc (3.29 mg/g), Rf (1.47 mg/g), Re (1.29 mg/g), and Rd (1.0 mg/g). The KRG tablets were made by dehydrating extracts of KRG. A total of 534 subjects who received a COVID-19 vaccine were recruited in August 2021 and continued with follow up. Subjects were assigned to two groups: the KRG group was taking KRG continuously before the study KRG per day for four weeks from enrollment, and the control group did not take KRG. In total, 174 subjects were included in the KRG group and 331 in the control group. Because of incomplete follow up or refusal of a third dose of COVID-19 vaccine administration, 155 subjects were excluded. Thus, there were 149 subjects in the KRG group and 201 in the control group. All subjects completed five visits (Figure 1).

### 2.2. Study Outcomes

The inclusion criteria for this study included the following: adults aged 30 years or older, participants who had taken red ginseng for at least one month within the last month prior to vaccination (KRG group), and individuals who had not taken red ginseng for two or more months before vaccination (control group). Subjects were excluded if they met any of the following criteria: patients being treated for cardiovascular diseases such as angina, myocardial infarction, stroke, uncontrolled diabetes, undergoing treatment for malignant tumors, taking antidepressants, antipsychotics, other psychiatric medications, and consuming functional foods including multivitamins. All subjects received the COVID-19 vaccine three times, and they visited the hospital five times to measure vaccine-induced antibodies to the spike protein receptor-binding domain (anti-S-Ab) and nucleocapsid (anti-NC) SARS-CoV-2 protein and anti-N-Ab. We checked anti-NC antibodies to evaluate whether patients were infected with COVID-19. No anti-NC antibodies were detected during or after vaccination. The second dose of the vaccine was administered four weeks after the first dose. We evaluated antibodies just before the second dose (baseline), 2, 4, and 12 weeks after the second dose, and 4 weeks after the third dose, independent of the type of vaccine. We first measured serum antibody levels and investigated possible changes at each time point. The change after 4 weeks compared with baseline was considered as the primary endpoint to calculate statistical power. We further compared changes in antibody levels in the KRG group vs. the control group.

### 2.3. Measurement of Anthropometric and Biochemical Parameters

Participants underwent five examinations: at baseline; 2, 4, and 12 weeks after their second COVID-19 vaccination, and 4 weeks after their third vaccination. At each visit, body weight, height, systolic blood pressure (SBP), and diastolic blood pressure (DBP) were recorded. Height and weight were obtained with subjects wearing light indoor clothing without shoes to the nearest 0.1 cm (Seca 225, Hamburg, Germany) and 0.1 kg, respectively (GL-6000-20, Gyeonggi, Republic of Korea). SBP and DBP values were measured three times in a seated position after a 5 min rest using a standard mercury sphygmomanometer (Baumanometer, Gresham, OR, USA). At baseline, we measured cholesterol metabolites and surveyed smoking status and alcohol consumption. Subjects were categorized into non-smoker and current smoker groups. Current drinkers were defined as those who drank alcohol more than once a month. We adopted binary variables of presence or absence for a history of hypertension and diabetes on a self-reported questionnaire. Body mass index (BMI) was calculated as the ratio of weight (kg)/height (m^2^). Blood samples were collected after >8 h of fasting. Fasting plasma glucose, total cholesterol, high-density lipoprotein (HDL) cholesterol, low-density lipoprotein (LDL) cholesterol, triglycerides, aspartate aminotransferase (AST), and alanine aminotransferase (ALT) were measured using the Cobas 8000 c702 module (Roche Diagnostics, Mannheim, Germany). White blood cell (WBC) count was measured using the XN-9000 (Sysmex Corporation, Kobe, Japan). A total of 25 hydroxyvitamin D (vitamin D) determinations were performed with the Cobas 8000 e801 module (Roche Diagnostics). Total immunoglobulin E (IgE, reference range, ≤100 kU/L) was tested with the Phadia 250 (Phadia, Uppsala, Sweden). Hypertension was defined by systolic blood pressure (SBP) ≥ 140 mmHg and diastolic blood pressure (DBP) ≥ 90 mmHg, or the current use of hypertension medicines. Type 2 diabetes mellitus (DM) was defined by a previous diagnosis of type 2 DM or a fasting plasma glucose level ≥ 126 mg/dL.

### 2.4. Detection of Virus-Specific Antibodies

Automated ECLIA tests were performed with two types of SARS-CoV-2 antibody kits using the Cobas 8000 e801 module (Roche Diagnostics). The Elecsys Anti-SARS-CoV-2 assay (Roche Diagnostics) uses a recombinant protein representing the nucleocapsid (NC) antigen for the qualitative detection of antibodies against SARS-CoV-2. Results with <1.0 cut-off indexes (COI) were interpreted as negative for anti-NC antibodies, and those with ≥1.0 were interpreted as positive for anti-NC antibodies. The Elecsys Anti-SARS-CoV-2 S assay (Roche Diagnostics) uses a recombinant protein representing the spike protein receptor-binding domain to quantitatively determine antibodies against SARS-CoV-2. Results with <0.80 U/mL were interpreted as negative for anti-S-Ab, and those ≥0.80 U/mL were interpreted as positive for anti-S-Ab. The surrogated virus neutralization tests (sVNT) were performed with a cPass SARS-CoV-2 Neutralization Antibody Detection kit (GenScript, Piscataway, NJ, USA) using the SpectraMax 190 (Molecular Devices, San Jose, CA, USA) based on ELISA to detect anti-N-Ab against SARS-CoV-2. The results were interpreted as percentage inhibition (%inhibition) based on OD450 intensity. The manufacturer-recommended cut-off of ≥30% signal reduction was used to indicate the presence of anti-N-Ab. All percent inhibition results were converted to IU/mL of the WHO International Standard using an Excel-based conversion tool [22]. The upper limit of the measurable range was 97.57% inhibition (or 3002 IU/mL). All tests were performed according to the manufacturer’s instructions.

### 2.5. Statistical Analysis

Data are shown as the means ± standard deviations for continuous variables and frequency (percentage) for categorical variables. Statistical significance for differences in baseline characteristics between the KRG and control groups was analyzed using independent *t*-tests for continuous. The linear regression was used to determine the difference of change compared with baseline between the KRG and control groups. We assessed the association between antibody level four weeks after the first dose (baseline) and other continuous variables using Pearson’s correlation analysis.

Longitudinal antibody data were analyzed using the linear trapezoidal rule to compare anti-N-Ab in KRG and control groups because different subjects were assessed at different times from vaccination. [23,24]. To compare the distribution of anti-N-Ab and for visual comparisons of distributions between different times in the two groups, the area under the curves (AUCs) using a linear trapezoidal rule is shown in. The PK package of R software was used for the AUC analysis [25].

## 3. Results

A total of 350 subjects who received three COVID-19 vaccines participated in this study. Subjects were allocated to the KRG group (*n* = 149) or control group (*n* = 201) depending on KRG use. Table 1 represents the baseline characteristics of the subjects in each group. There were differences in age, sex, AST, and vitamin D between the two groups. The KRG and control groups were similar in BMI and hypertension. There were no differences in fasting plasma glucose, lipid profiles, ALT, and Ig E between the two groups (Table 1).

Pearson’s correlation analysis was used to evaluate the association of anti-N-Ab and other covariates four weeks after the first dose (baseline). Age and vitamin D level were inversely correlated with anti-N-Ab (Age: r = −0.279, *p* = 0.001; vitamin D: r = −0.147, *p* = 0.001), and WBC count and anti-S-Ab were significantly correlated with anti-N-Ab (WBC: r = 0.097, *p* = 0.028; anti-S-Ab: r = 0.736, *p* = 0.001) (Table 2).

Common patterns of antibody response are shown in Table 3. The first dose of a vaccine-induced production of anti-S-Ab and anti-N-Ab and the second dose caused a significant increase in anti-S-Ab for two weeks. A decrease in antibodies after the peak was observed until 12 weeks after the second dose, and then, after booster vaccination, anti-S-Ab and anti-N-Ab were increased again.

**Figure 2:**
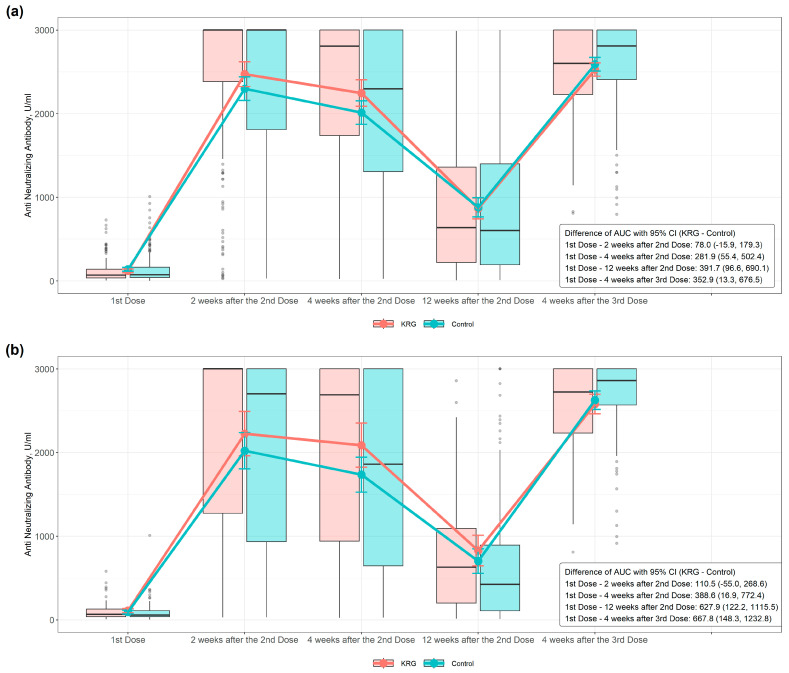
Antibody responses to two vaccine doses in two groups: (**a**) Anti-Neutralizing-Ab in subjects and (**b**) Anti-Neutralizing-Ab in subjects over 50 years old. Blood samples were collected from individuals 4 weeks after first dose; 2 weeks, 4 weeks, and 12 weeks after second dose; and 4 weeks after the third dose. Neutralizing antibodies were screened and differentiated using the Neutralization Antibody Detection kit as described in the Materials and Methods. The x-axis shows time points just before the second dose of vaccine administration; 2, 4, and 12 weeks after the second dose; and 4 weeks after the third dose. Meanwhile, the y-axis shows antibody concentrations as mean ± SD. The area under the curve (AUC) was calculated using a linear trapezoidal rule.

AUCs using a linear trapezoidal rule revealed statistically significant differences between the two groups for changes in anti-N-Ab over time. Figure 2 summarizes these results for each time point. We found that the antibody formation rate of anti-N-Ab in the KRG group decreased more slowly than in the control group after the second vaccination, and this trend was maintained, with a significantly higher level than that of the control group, between the first dose and four weeks after third vaccination (difference of AUC, 352.9, 95% CI 7.5–698.4) (Figure 2a). Subgroup analyses showed a significant difference in AUC of antibody formation rate between the KRG and the control group between the first dose and four weeks after the second dose in subjects aged 50 years or older (−76.7, 95% CI −225.8–252.6) (Figure 2b).

## 4. Discussion

In this study, vaccine-induced humoral responses in both the KRG and control groups significantly increased two weeks after the second administration of all vaccines; then, a decrease was observed until 12 weeks or just before booster vaccination. However, after booster vaccination, humoral responses increased again; this trend was similar to the data in previous studies [26]. The antibody formation rate of the KRG group was sustained higher than that of the control group throughout the study period. In particular, this trend was prominently observed in those over 50 years old.

Poor vaccine responses, such as reduced humoral responses and delayed T-cell responses after vaccination, including COVID-19 vaccines, in the elderly due to immunosenescence are well established [27,28,29,30]. In our study, a negative correlation between anti-N-Ab titers and age was observed, similar to the results of previous studies [31,32]. However, the antibody formation rate of the KRG group was higher than that of the control group, suggesting that KRG plays a positive role as a COVID-19 vaccine adjuvant and can improve the immune response elicited by the COVID-19 vaccine. Saponins have been studied as adjuvants in vaccines that enhance the adaptive immune response to a vaccine because of their ability to improve the innate immune response and humoral and cellular immunity [33]. These studies suggested the possibility of saponin-based adjuvants in COVID-19 vaccines inducing effective immunity in the elderly with a decline in cell-mediated immunity [34,35]. In a clinical study of varicella-zoster virus vaccine using saponin, vaccine effectiveness was over 90% in persons over 50 [36].

There are several possible factors related to this unique outcome regarding the maintenance of a higher vaccine-induced humoral response in the KRG group. First, KRG can help to enhance the effectiveness of COVID-19 vaccines through cytokine and chemokine regulation and immune cell proliferation and activity increase. Chemokines and cytokines are critical factors in innate immunity and inflammation, and they play essential roles in developing and maintaining adaptive immunity in response to vaccination [37]. Christina et al. [38] reported that the COVID-19 vaccine-related cytokine response induces IFN-r, IL-15, and IP-10/CSCL10, which play a pivotal role in stimulating innate immune responses, forming adaptive immunity, and eliciting immunological memory. After the second dose of the COVID-19 vaccine, these cytokines also induced TNF-α and IL-6. In the same study, changes in INF-r and IL-15 levels were confirmed to be positively correlated with COVID-19 vaccine-induced antibody titers, and the authors suggested that this indicated the development of an effective humoral response after vaccination. KRG improves the body’s immune reaction by regulating the secretion of cytokines that mediate the immune response [12,14,39]. Some studies reported that KRG enhances immunomodulatory effects by improving the production of NO and pro-inflammatory cytokines, including TNF-α and IL-6 [40,41].

COVID-19 vaccination also induces SARS-CoV-2-specific CD8+ and CD4+ T cell responses, which might confer long-term immune memory against SARS-CoV-2 [42,43]. Sahin et al. [43] reported concurrent production of COVID-19 vaccine-induced neutralizing antibodies and activation of virus-specific CD8+ and CD4+ T cells seven days after the second vaccination, but CD4+ T cell responses were not detectable at baseline, and immunomodulatory cytokines such as IFN-r were released by CD8+ T cells. These results indicate a favorable cellular immune response with antiviral and immune-enhancing properties that strongly complement the neutralizing antibody response. KRG also stimulates host immunity by increasing the activity and number of T cells and Β cells and increasing the number of WBCs [13,39,44,45]. Suh et al. [46] reported that patients with decreased immunity underwent surgery for gastrointestinal cancer and found increased CD8+ T cells, CD4+ T cells, and WBCs, and IL-2 in the blood enhanced immune function after cancer surgery in the group taking KRG. Studies in animal models have reported that KRG improves immunity by raising the number of WBCs in the blood [13,47]. In addition, a clinical study on healthy subjects reported that KRG helped improve immunity with an increase in the number of immune cells, especially T cells, Β cells, and WBCs, after eight weeks of using KRG [13]. In animal experiments, daily oral administration of KRG in combination with vaccination significantly increased anti-influenza virus antibody titers and decreased the frequency of influenza symptoms [21]. In our study, the number of WBCs increased, and anti-N-Ab titers had a positive correlation with the number of WBCs in the KRG group, and at four weeks after the second vaccination, the KRG group maintained a higher anti-N-Ab ratio than the control group. These results strongly support the hypothesis that KRG helps to enhance humoral immunity after COVID-19 vaccination. In the future, more detail will be needed to study the effects and mechanisms of KRG’s effects on immune responses such as T cells, B cells, and cytokines after COVID-19 vaccination.

We further examined the effect of the micronutrients in KRG. Previous studies showed that the host’s nutritional status influences immune response, and recent comprehensive review papers demonstrated the importance of individual micronutrients for immune response and the various mechanisms of action [48,49]. KRG contains numerous nutritional components, including glycoside-containing saponins; nitrogen-containing complex proteins; alkaloids; phenolic compounds; nucleic acids; amino acids; essential oils; fat-soluble fatty acids; polyacetylenes; phytosterols; terpenoids; saccharides including monosaccharides, oligosaccharides, and polysaccharides; pectin substances; vitamin B complex; and minerals (nickel, aluminum, vanadium, phosphorus, cobalt, manganese, germanium, and copper) [39,50,51]. These various micronutrients in KRG might help strengthen immunity. However, in our study, vitamin D concentration and anti-N-Ab titers were negatively correlated. However, the average vitamin D concentration of the participants was only about 20 ng/mL, which is less than the recently reported optimal level (40–60 ng/mL) for achieving immune system enhancement and health benefits [52,53]. However, a systematic review and meta-analysis of nine studies involving 2367 patients showed that seroprotection against influenza A virus subtype H3N2 and influenza B virus was lower in those with vitamin D deficiency [54]. In a population-based intervention study in Spain, vitamin D treatment with 23(OH)D concentrations of 30 ng/mL or higher successfully reduced the risk of SARS-CoV-2 infection, long COVID-19 symptoms, and death [55].

Conversely, Chillon et al. [56] also reported no significant difference according to the concentration of vitamin D in vaccine-induced SARS-CoV-2 IgG concentration and anti-N-Ab. Thus, further research is needed to evaluate the effect of vitamin D on COVID-19 vaccination. Additionally, based on the results of several clinical trials for the safety of KRG in healthy Korean adults [13,41,57], the safety of taking 3 g KRG per day for four weeks in our study was validated.

This study has several limitations. First, the participants in the KRG group were those who took KRG per day for four weeks or were taking KRG continuously. For those taking KRG continuously, it was difficult to determine the dosage of KRG accurately. However, continuous KRG intake was checked at each visit through a questionnaire. The KRG group maintained a relatively high vaccine-induced immune response compared with the control group at the end of this study, suggesting that KRG provides sufficient vaccine benefits at an indirect level. Second, during the clinical trial period, to minimize the effects of nutrients other than KRG, we told participants to stop all nutritional supplements, including vitamin D, which could affect vaccine-induced immune responses. Although nutritional supplement intake was checked through a questionnaire at each visit, the possibility of unobserved bias could not be excluded because it was a self-reported questionnaire. Third, vaccines based on different immunogenicity principles were included, including mRNA-based vaccines (Pfizer and Moderna) and recombinant adenoviral vector vaccines (AstraZeneca). However, this study was not a randomized clinical trial comparing antibody titers and KRG intake between vaccines. Regardless of KRG intake, the overall vaccine effectiveness of our study was confirmed to be consistent with the results of the previous studies, in which the antibody titers peaked two weeks after the second vaccination and then decreased after that [4,58,59]. Fourth, during the study period, subjects were not recruited as much as the required sample size and likely was underpowered. Nevertheless, significant results were obtained for the primary Anti-N-Ab difference of change after 4 weeks compared with baseline.

Moreover, the study population could not choose a vaccine and were given vaccines according to the vaccine protocols defined by the Korean government (KDCC). This study was performed in a single center; however, our study had the benefit of observing the same subjects longitudinally. Last, the effect of KRG on cellular immunity after COVID-19 vaccination was not tested; however, KRG modulated or enhanced immune response in previous in vitro and in vivo studies [11,39], as well as in clinical studies [13,17,46]. The strength of this study is that it was the first clinical study on the effect of KRG on the humoral immune response to COVID-19 vaccination.

## 5. Conclusions

The protection against COVID-19 tends to decline over time after vaccination. This study showed the potential of KRG for boosting immunity and helping maintain a higher vaccine-induced humoral response after COVID-19 vaccination. Further studies will be needed to explain the mechanisms of these relationships in detail.

## Figures and Tables

**Table 1:** Baseline characteristics^*^ of the study population according to KRG intake.

Variables		KRG ^1^ (N = 149)	Control (N = 201)	*p*-Value
Age, years		47.2 ± 9.0	50.3 ± 9.8	0.002 *
Sex (male, %)		104 (69.8)	80 (39.4)	0.001
Body mass index		24.5 ± 3.2	24.6 ± 4.0	0.814 *
Glucose, mg/dL		99.8 ± 19.1	102.2 ± 22.0	0.285 *
Total Cholesterol, mg/dL		191.6 ± 37.8	177.8 ± 37.8	0.986 *
Triglyceride, mg/dL		116.0 (74.0–173.0)	109.0 (72.0–165.0)	0.193
HDL ^2^ cholesterol, mg/dL		55.2 ± 16.3	54.1 ± 14.5	0.539 *
LDL ^3^ cholesterol, mg/dL		123.9 ± 35.6	111.1 ± 35.4	0.001 *
Ig E		65.7 (21.1–165.0)	46.0 (18.5–102.0)	0.152
Vitamin D		22.5 ± 9.14	24.1 ± 11.0	0.017 *
White blood cells (×10^3^ L)		5.830 ± 1.38	6.081 ± 1.541	0.153 *
AST ^4^ (IU/L)		24.2 ± 20.1	23.5 ± 14.2	0.001 *
ALT ^5^ (IU/L)		24.8 ± 15.4	23.7 ± 16.6	0.343 *
Hypertension, (%)		25 (16.1)	46 (22.9)	0.152
Diabetes, (%)		6 (4.0)	31 (15.4)	0.001
First dose	Second dose			0.027
Az ^6^	Az ^6^	17 (11.4)	36 (17.9)	-
Az ^6^	Pfizer	22 (14.8)	15 (7.5)	-
Moderna	Moderna	40 (26.8)	41 (20.4)	-
Pfizer	Pfizer	70 (47.0)	109 (54.2)	-

^1^ Korean Red Ginseng, ^2^ high-density lipoprotein, ^3^ low-density lipoprotein, ^4^ aspartate aminotransferase, ^5^ alanine transaminase, ^6^ AstraZeneca. * Data are expressed as mean ± SD or percentage, and *p*-values were calculated using ANOVA or the chi-square test.

**Table 2:** Correlations between baseline anti-neutralizing antibody (Anti-N-Ab) and other variables.

Variables	Subjects	
	r ^1^	*p*-Value *
Age, years	−0.279	0.001
Body mass index	−0.016	0.715
Glucose, mg/dL	0.022	0.617
Total cholesterol, mg/dL	0.038	0.388
Triglyceride, mg/dL	−0.021	0.635
HDL ^2^ cholesterol, mg/dL	0.017	0.689
LDL ^3^ cholesterol, mg/dL	0.029	0.505
Ig E	0.010	0.822
Vitamin D	−0.147	0.001
White Blood Cells (×10^3^ L)	0.097	0.028
Anti-S-Ab ^4^	0.736	0.001
AST ^5^ (IU/L)	0.078	0.078
ALT ^6^ (IU/L)	0.066	0.133

^1^ Correlation coefficient, ^2^ high-density lipoprotein, ^3^ low-density lipoprotein, ^4^ anti-surface antibody, ^5^ aspartate aminotransferase, ^6^ alanine transaminase. * *p*-values were calculated using Pearson’s correlation analysis.

**Table 3:** COVID-19 antibody after vaccination in the KRG and control groups.

COVID-19 Antibody (Anti-N-Ab ^2^)	KRG ^1^ (N = 149)	Control (N = 201)	*p*-Value *
2 weeks after the second dose—first dose (baseline)	2350.74 ± 898.48	2160.30 ± 995.34	0.042
4 weeks after the second dose—first dose (baseline)	2125.56 ± 949.85	1874.02 ± 989.24	0.014
12 weeks after the second dose—first dose (baseline)	745.39 ± 709.05	742.81 ± 770.13	0.739
4 weeks after the third dose— first dose (baseline)	2405.77 ± 512.71	2451.61 ± 611.84	0.796

^1^ Korean Red Ginseng, ^2^ anti-neutralizing antibody. * *p*-values were calculated based on a linear regression to adjust for age, sex, and vaccine type.

## Data Availability

All data generated or analyzed during this study are available from the corresponding author upon proper request.
